# SERS Effect on Spin-Coated Seeding of Tilted Au-ZnO Nanorods for Low-Cost Diagnosis

**DOI:** 10.3390/ma13235321

**Published:** 2020-11-24

**Authors:** Miyeon Jue, Chan-Gi Pack, Seakhee Oh, Bjorn Paulson, Kwanhee Lee, Jun Ki Kim

**Affiliations:** 1 Asan Institute for Life Science, Asan Medical Center, Seoul 05505, Korea; imascai@naver.com (M.J.); changipack@amc.seoul.kr (C.-G.P.); bjorn.paulson+mtrls@gmail.com (B.P.); gtgay@naver.com (K.L.); 2 Department of Convergence Medicine, University of Ulsan College of Medicine, Seoul 05505, Korea; 3 Department of Pediatrics, Asan Medical Center Children’s Hospital, University of Ulsan College of Medicine, Seoul 05505, Korea; seakhee.oh@amc.seoul.kr

**Keywords:** ZnO nanorods, Raman spectroscopy, finite element method, optical diagnosis, label-free diagnosis, surface-enhanced Raman spectroscopy, local surface plasmon resonance

## Abstract

Uniformly parallel Au-coated ZnO nanorods have previously been shown to amplify local Raman signals, providing increased sensitivity to disease markers in the detection of inflammation and cancer. However, practical and cost-effective fabrication methods of substrates for surface-enhanced Raman spectroscopy (SERS) fail to produce highly uniform surfaces. Here, the feasibility of Raman enhancement on less-uniform substrates is assessed. ZnO nanorod structures were fabricated by hydrothermal synthesis, starting from spin-coated seed substrates. Following analysis, the nanostructures were coated with Au to create stochastically variant substrates. The non-uniformity of the fabricated Au-coated ZnO nanorod structures is confirmed morphologically by FE-SEM and structurally by X-ray diffraction, and characterized by the angular distributions of the nanorods. Monte Carlo finite element method simulations matching the measured angular distributions and separations predicted only moderate increases in the overall Raman enhancement with increasing uniformity. Highly variant substrates exhibited approximately 76% of the Raman enhancement of more uniform substrates in simulations and experiments. The findings suggest that, although highly inhomogeneous Au-coated ZnO nanorod substrates may not attain the same Raman enhancement as more uniform substrates, the relaxation of fabrication tolerances may be economically viable.

## 1. Introduction

The efficient and reliable detection of trace biomarkers to identify tissue and organ pathologies remains a challenge in physiology, nutrition, toxicology, and theranostics [1,2,3,4]. In surface-enhanced Raman spectroscopy (SERS), nanostructured materials are used to enhance Raman spectra for the detection of target analytes. Compared to fluorescence-based methods, SERS promises label-free and non-invasive diagnoses. By measuring proteins, lipids, nucleic acids, exosomes, metabolites, and other nanoscale biological molecules directly as they appear (or fail to appear) in common biological fluids, information about cancer, inflammation, or other diseases can be gathered [5,6,7].

Surface-enhanced Raman spectroscopy facilitates the inelastic scattering of incident electromagnetic waves by the target bio-sample, revealing the vibrational fingerprint of the target molecules. Although the contribution of electrical and chemical phenomena in SERS was somewhat disputed [8,9], localized surface plasmon resonances are now thought to account for the majority of local enhancements [9]. The SERS effect is commonly characterized by a Raman enhancement factor (EF), which quantifies the magnitude of the SERS signal increase compared to that of a non-structured substrate [10]. The EF has been shown to strongly depend on a number of factors—particularly the gaps between the metallic particles that support plasmon formation—through generalized multisphere Mie scattering and finite element method (FEM) simulations [8]. For this reason, the majority of SERS experiments have either used highly uniform, nanostructured substrates or colloidal solutions that exhibit only a stochastic structure. Currently, the techniques for the fabrication of highly uniform SERS substrates are limited to lithography and single-crystal growth. Several groups have sought to reduce the cost of SERS substrates while increasing large-area substrate uniformity [11]. These techniques have been focused on tilings of low-dimensional structure. Disordered substrates have also been characterized experimentally. Shi et al. presented a nanowire-based substrate able to detect phorate and melamine at concentrations of 0.1 μg·mL^−1^ because of the presence of randomly generated enhancement hotspots [12]. Eshkeiti et al. pioneered the inkjet printing of nanoparticle-based SERS substrates on Si wafers, obtaining EFs of 3~5; however, their surface exhibited wide-area non-uniformity (as determined by profilometry) with a corresponding non-uniformity of the SERS signal [13]. More recently, several groups have demonstrated improved inkjet printing, achieving consistent EFs greater than 10^4^ [14].

The development of SERS for medical applications requires the development of cost-effective substrates. ZnO has been demonstrated to exhibit a wide variety of desirable geometries, such as hierarchical nanostructures, nanobelts, and nanohelices [15,16,17,18]. In particular, the solution-based self-assembly of ZnO nanorods provides a means for increasing the uniformity of low-cost fabrication methods: nanorods are grown from single- or polycrystalline substrates, which provide initial nucleation sites for crystal growth. At the nanoscale, crystal growth and alignment are guided by interfacial tension and limited by the precursor concentration in the carrier solution. Nanorods are favored for uptake of ZnO, provided the local precursor concentration is not depleted by nearby nanorods (a selection process for existing nanorods, which may be exacerbated by Ostwald ripening). Thus, nanorods fabricated from ZnO solutions result in SERS substrates that exhibit selectivity toward relatively uniform inter-rod gaps, which is useful for biological applications [19,20]. Uniform ZnO substrates coated with Au have enabled NIR SERS while also providing cellular non-toxicity [19].

In this study, low-cost SERS substrates based on Au-coated ZnO nanorods were fabricated in varying orders by spin-coating of a seed layer prior to the ZnO nanorod hydrothermal growth phase. Morphological assessment by scanning electron microscopy (SEM) and X-ray diffraction (XRD) prior to Au nanoparticle ripening revealed that while the spacing of nanorods on the substrate was relatively consistent, while the distribution of tilt angles varied significantly compared to that reported in a previous work on uniform ZnO substrates. Experimental verification of the Raman EF showed an ~1.3 times decrease in the most severely disordered substrates, which is satisfactory for Raman spectroscopy. A Monte Carlo FEM analysis of a randomly generated two-dimensional nanorod distribution resembling the fabricated Raman substrate revealed that the observed dependence of EF on fabrication parameters was due to the inherent uniform spacing of the nanorod seeds, caused by a kinetic process. It is concluded that less-ordered substrates, while not satisfactory for single-molecule SERS or high-performance SERS applications, can be modeled by FEM to analyze their behavior for future low-cost SERS substrate designs.

## 2. Materials and Methods

**Figure 1:**
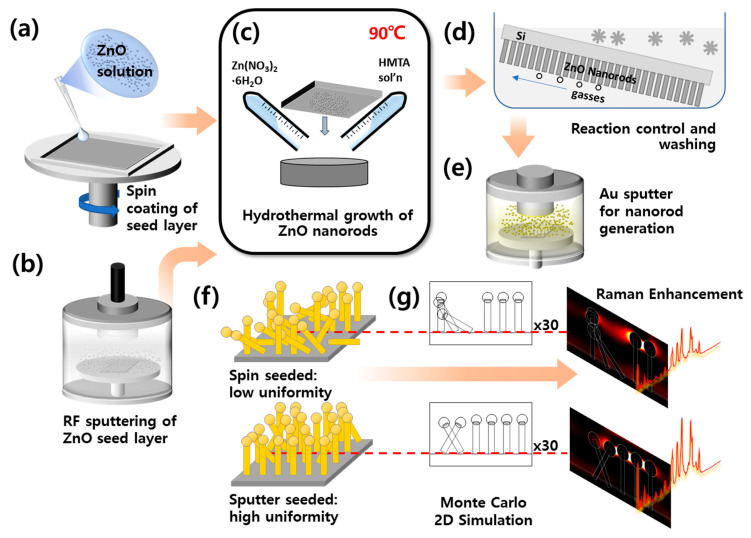
Schematic of the experiment. (**a**) Randomly tilted ZnO nanorods seeded by spin-coating, and (**b**) highly uniform ZnO nanorods seeded by sputtering on Si substrates. (**c**) ZnO grown by a hydrothermal process from zinc nitrate and hexamethylenetetramine (HMTA) solutions. (**d**) Controlled tilt applied to substrates allowing reaction gases to disperse. (**e**) Au-coating by sputtering, leading to plasmonic effects. (**f**) Spun substrates with lower uniformity. (**g**) Raman enhancement in simulations and experiment.

A spin-coating process based on solution transfer was used to initiate the low-cost seeding of ZnO nanorods. A Si wafer was cleaned and cut to a size of 1 × 1 cm^2^. A precursor seed solution of 20 mM zinc acetate dihydrate (Zn(CH_3_COO)_2_·2H_2_O), ≥99.0%, Sigma-Aldrich, St. Louis, MO, USA) in 2-propanol was prepared. Following sonication, the seed solution was deposited dropwise onto the 1 × 1 cm^2^ substrates. The substrate was spin-coated 10 times at 3000 rpm for 30 s each. This process is shown schematically in Figure 1a. The substrates were removed from the spin-coater and annealed at 350 °C for 20 min in air. Seeded samples were subsequently grown by hydrothermal synthesis as discussed below.

To generate high-uniformity controls, sputter-seeded ZnO nanorod samples were fabricated following the sputtering protocol reported in previous studies [6,19,20], wherein a 30 nm organic seed layer was deposited by radio frequency (RF) magnetron sputtering at 100 W power for 30 min, as shown schematically in Figure 1b.

Following seeding, spun and sputtered seed samples were grown by identical hydrothermal synthesis processes. ZnO nanorods were grown from a solution of 25 mM zinc nitrate hexahydrate (Zn(NO_3_)_2_·6H_2_O, ≥99.998%, Alfa Aesar, Ward Hill, MA, USA) and 25 mM hexamethylenetetramine (HMTA ≥ 99.5%, Sigma-Aldrich) in 50 mL deionized (DI) water heated at 90 °C for 60 min (Figure 1c). The substrates were kept inclined in the solution to facilitate the removal of oxygen (which acts as a hindrance to nanorod growth) (Figure 1d), and then washed with DI water and dried with nitrogen gas.

The grown ZnO nanorod structures were characterized by field emission SEM (FE-SEM) and XRD. The XRD data for the ZnO nanorod substrates were acquired using an X’Pert PRO X-ray diffractometer (PANalytical B.V., Almelo, The Netherlands) at room temperature with a 2 kW Cu target at 45 kV × 40 mA.

Thermally evaporated Au deposition on the nanorod substrates was performed using an ion coater (Hoyeon Tech Co., Ltd., Seongnam, Korea) (Figure 1e). A deposition rate of 4.55 nm·min^−1^ was used for 11 min at 0.1 mbar bare pressure and 25 mA deposition current. The nanorod substrates were coated with an amount of Au equivalent to a thin film of 200 nm thickness [19]. The Au deposition was assessed by FE-SEM cross-sections (S-4700, HITACHI, Tokyo, Japan) acquired at 10 kV beam voltage.

Two-dimensional FEM simulations were performed in COMSOL Multiphysics v. 4.2 (COMSOL Inc., Burlington, MA, USA). Nanorod-like structures were created with the built-in parametrized geometry primitives, and the materials were assigned manually. A domain of five times the area of interest in width (*x* or *y*) and four times the nanorod length in height (*z*) was found to minimize the effect of domain size on the simulation results when scattering boundary conditions were used. Raman excitation was simulated by a plane wave (785 nm) linearly polarized along the horizontal and incident on the top surface of the 2D domain. The permittivity of Au was set to ε_Au_ = −22.855 + 1.425i at 785 nm [21,22], while the refractive index of ZnO was calculated from the dispersion curve of Stelling [23] to be 1.6014 at 785 nm. The refractive index of the analyte was assumed to be unity, as was that of the ambient air.

For pairwise simulations, a single pair of nanorods was simulated, separated by 177 nm at their bases and tilted at representative angles. The resulting Raman intensities were weighted based on the observed nanorod angular distribution for each seed condition. A total of 128 pairwise simulations were performed.

For Monte Carlo simulations, a 3D geometry of nanorods tiled 5 × 5 across a plane was projected into the 2D simulation domains (*x-z* and *y-z*). Azimuthal and polar angles were generated for each nanorod using uniform pseudorandom generators in Python 3.7 to match the observed nanorod angular distribution, saved for future reproducibility, and exported to COMSOL Multiphysics. Tilted nanorods were simulated in sets of five at 177 nm base separations, and 60 Monte Carlo simulations were performed, with 15 for each combination of seed conditions and orthogonal 2D projection (spin/sputter and *x-z*/*y-z*).

A model analyte of 1 mM rhodamine B (RhB; Junsei Chemical Co., Ltd., Tokyo, Japan) in water was prepared based on a previously reported procedure [20], wherein 5 μL drops of the solution were deposited onto the SERS chips and dried prior to Raman spectroscopy. Illumination conditions remained uniform over all samples. Illumination was provided by a 785 nm diode laser via a 1.45 NA 100× objective lens, yielding a beam spot diameter of approximately 1 μm. The spectra of the samples were measured from 10~12 points at room temperature across a range of 550–1800 cm^−1^ with integration time of 15 s. The Raman spectra were corrected for the fluorescence of RhB by applying 3rd-order polynomial fitting and Savitzky-Golay smoothing using RAON-Spec (NOST, Seongnam, Korea). Spectral visualizations were plotted in Origin 2018b (OriginLab Corp., Northampton, MA, USA).

Due to difficulties in correcting for analyte concentrations in 3D structures [24], we apply a measurement for EF which is independent of the volume distribution of analyte. To correct for effective analyte concentrations, the EFs of the experimental data were calculated relative to the measured Raman spectra of RhB on the bare Si substrate, with a correction for concentration due to the droplet dispersion on the sample chip by the following equation:

(1) $$\mathrm{EF}=\left(\frac{I_{\mathrm{SERS}}}{I_{\mathrm{bare}}}\right)\left(\frac{c_{\mathrm{bare}}}{c_{\mathrm{SERS}}}\right)$$

where $I_{\mathrm{SERS}}$ is the Raman intensity from RhB on the Au-coated nanorod substrate and $c_{\mathrm{SERS}}$ is the concentration of analyte on the substrate. Likewise, $c_{\mathrm{bare}}$ is the concentration of analyte on a sample of the ZnO nanorod substrate without gold deposited, and $I_{\mathrm{bare}}$ is the corresponding measured Raman intensity [19]. Different analytes concentrations were used for each substrate due to difficulty in detecting signals from the bare substrates. Neither bare ZnO nor ZnO-Au substrates displayed coffee ring effects [19] when RhB was deposited. Rhodamine B was observed to spread nearly equally between the bare ZnO substrates and gold-coated ZnO substrates in both the spin-seeded and sputter-seeded cases, with the effective deposition area of single RhB drops being 7.3 and 11.6 mm^2^, respectively. An RhB concentration of 100 mM was deposited for “bare” substrates, and a concentration of 1 mM was used for gold-coated substrates. All other measurement conditions, including laser power, laser spot size, and integration time were kept the same for all measurements. It should be noted that this differs from a more classical EF [25].

## 3. Results

### 3.1. Fabrication of Tilted Nanorods and Characterization of Nanorod Morphology

The spin-coating technique used for the seeding of ZnO on Si resulted in low-cost SERS substrates. Optical microscopy revealed nanorods distributed on the 10 × 10 mm^2^ sample consistently with few micron-scale surface irregularities. Crystal orientation was measured across the entire ZnO nanorod sample by XRD prior to Au deposition.

**Figure 2:**
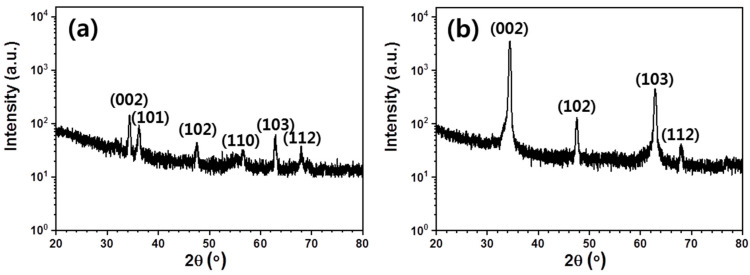
XRD data for (**a**) solution-seeded ZnO nanorods, and (**b**) sputter-seeded ZnO nanorods. The distribution of diffraction planes is more varied in sputter-seeded nanorods. Peaks are labeled with their corresponding lattice indices. The areas under each labeled peak were fit with Gaussians to determine the distribution of rod angles.

The distribution of diffraction planes offers an important characterization of the nanoscale structural order. It is known that ZnO nanorods exhibit a hexagonal wurtzite crystal structure [26]. As each individual ZnO nanorod constitutes a single crystal with a self-consistent structure, the XRD peaks are representative of crystal plane orientations relative to the substrate. The reflected XRD intensity distribution can be used to derive the distribution of absolute nanorod angles from the normal substrate (“vertical”). The XRD spectra in Figure 2 show several sharp diffraction peaks for each sample, representative of a discrete distribution of crystal structure angles. The amorphous background signal is representative of previous sol-deposited ZnO layers on Si substrates [27]. The distribution of angles was calculated from Figure 2 and is presented in Table 1.

In the solution-seeded sample, approximately 35% of nanorods exhibited orientations (101) and (110), corresponding to incident angles of 60° and 30° relative to the normal substrate, respectively. These orientations were not significantly detected in the more uniform sputter-seeded sample. It should be noted that the XRD signal strength is proportional to the volume of nanorods at a particular orientation, rather than to an absolute number. The observed distribution approximates the orientation distribution of nanorods (as counted), provided there exists no correlation between nanorod orientation and length.

**Figure 3:**
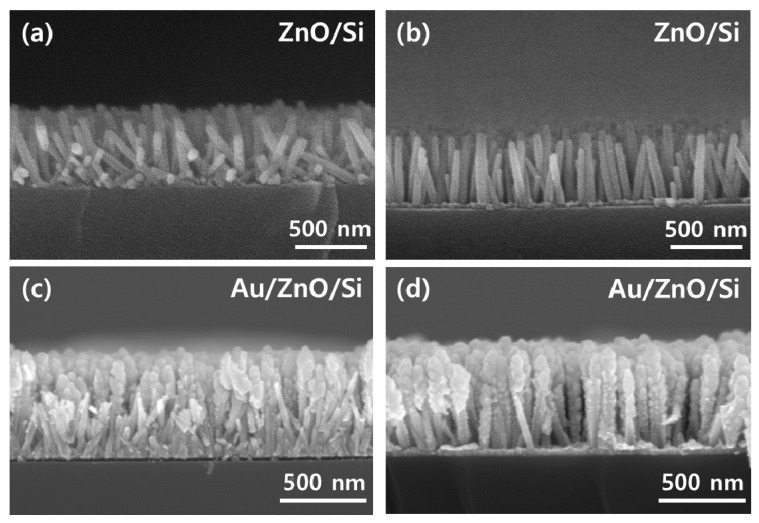
Cross-sectional SEM morphology of the fabricated nanorod structures: (**a**) spin-seeded nanorods before Au deposition, (**b**) sputter-seeded nanorods before Au deposition, (**c**) spin-seeded nanorods after Au deposition, (**d**) sputter-seeded nanorods after Au deposition.

As shown in Figure 3, SEM images of short (2.53 μm) sections of the ZnO-covered chips revealed that the nanorod length did not differ significantly between the two growth conditions for upright nanorods, justifying the use of the XRD area as a proxy for the nanorod distribution. Furthermore, upon examination of the nanorods in the SEM images, it was found that the nanorod tilt did not have a significantly favored direction (left/right) in either sample. The rate at which nanorods were obscured by other nanorods in SEM images was used to estimate the separation between nanorods for the sputter case (Table A1 in Appendix A), providing an approximate density of 5.5 nanorods μm^−1^ in the sectioned plane and 30.6 rods μm^−2^ as a function of area. For the solution-seeded sample, the high amount of occultation (including sets of mutually obscuring nanorods) made this strategy less accurate, and the seed density was assumed to be the same for both sample types.

### 3.2. Pairwise Finite Element Method Simulations

**Figure 4:**
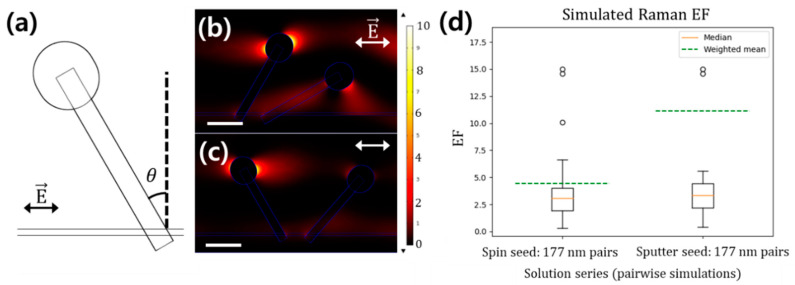
Comparison of simulated enhancement factors (EFs) derived by weighing pairwise Au-covered ZnO nanorod finite element method (FEM) calculations tilted based on their expected frequencies in the spin-seeded and sputter-seeded samples. (**a**) Nanorod showing its normal angle to the surface. (**b**) Pair of nanorods with angles 30° and 60°, scale bars 200 nm. (**c**) Pair of nanorods with angles −30° and 30°, scale bars 200 nm. Arrows depict the incident electric field polarization. (**d**) Distribution of simulated enhancement factor for the nanorod pairs. Averages weighted by the experimental angular distribution are shown across each boxplot as a dashed green line. (N = 128).

Computational FEM analysis was performed to understand the effect of non-uniform deposition on SERS enhancement. Structures were modeled based on the data from SEM images, with heads of 80 nm radius on nanorods with a width of 50 nm and length of 450 nm. An additional 0.45 nm structure was created around each nanorod to simulate the region of analyte adherence; this was found to increase simulation consistency, possibly by forcing high-density mesh formation at the nanorod surface. Au domains touching the analyte were rounded off to avoid hotspot formation. Initially, nanorods with angles selected from the set observed by XRD were simulated pairwise on a 2D plane at fixed separations. The observed distributions of local field enhancement are shown in Figure 4. Although the angular distributions are largely overlapping, when the results were weighted based on the angular distribution from Table 1, an EF reduction of 2.5 times was predicted for the spin-seeded substrates. These weighted EFs are shown as green dashed lines in Figure 4d. The behavior of the weighted mean EF is strongly determined by a single case: visible as the highest outlier in Figure 4d, nanorods with angles 0° and 0° have the highest EF and constitute 10% of expected pairs on the spin-seeded substrate, while constituting 69% of the expected pairs on the sputter-seeded substrate. All else being equal, the second most common pairing, with angles of 30° and 0° comprises 4.9% of spin-seeded pairs and 10% of sputter-seed pairs, and exhibits about 5× lower EF on a per-nanorod basis. The 72° and 72° pairing is the second-highest EF nanorod pair, visible as the second-highest outlier in Figure 4d, but constitutes less than 0.4% of the nanorod pairs on each substrate.

### 3.3. Monte Carlo Method for Simulation of EFs

**Figure 5:**
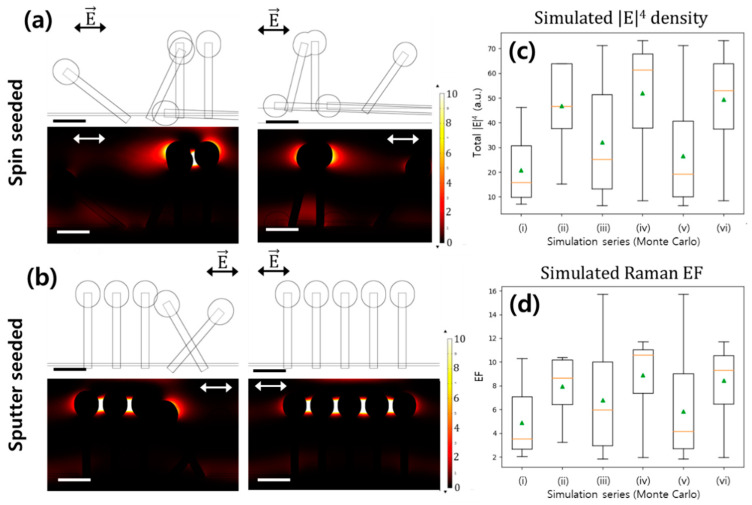
Monte Carlo simulations of Au-coated ZnO nanorod structures. Structures and simulated |E|^4^ for two randomly generated (**a**) spin-seeded and (**b**) sputter-seeded substrates. (**c**) Comparison of total magnitude |E|^4^ between spin-seeded and sputter-seeded simulations. (**d**) Comparison of EF between spin-seeded and sputter-seeded simulations. (i) *x-z* projection of spin-seeded ZnO-Au nanoparticles (ii) *x-z* projection of sputter-seeded NPs (iii) *y-z* projection of spin-seeded NPs (iv) *y-z* projection of sputter-seeded NPs (v) combining both projections of spin-seeded NPs (vi) combining both projections of sputter-seeded NPs. Green triangles represent arithmetic means; orange lines represent medians. Scale bars, 200 nm.

In order to model more complicated structures, the Monte Carlo scheme was employed to simulate the interactions between the nanorods. Two-dimensional arrays of five nanorods each were simulated according to the physical parameters measured above. The simulated nanorod angle distribution followed the angular distribution obtained by XRD; however, the angles were projected onto the 2D plane of simulation. In the case of occlusion, the nanorod with the higher angle was truncated to simulate the observed pattern of ZnO growth. Combining repetitions under each of two orthogonal projection conditions, a total of 30 simulations were performed for each seed condition to accurately capture the substrate statistics. The distribution of the observed SERS enhancement is plotted in Figure 5. Although the SERS effect is dominated by outliers, especially in the case of the less-uniform substrate, the simulation accurately predicts an adequate SERS EF from both substrates.

### 3.4. Experimental EFs

**Figure 6:**
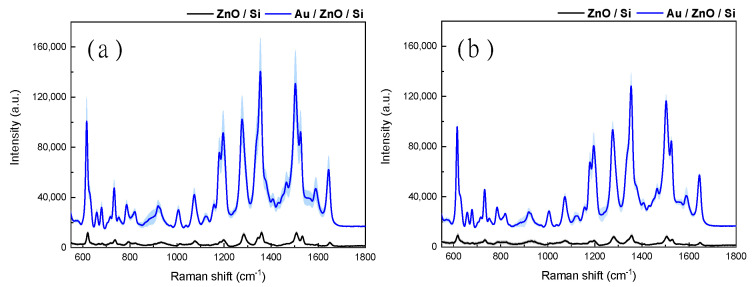
Raman spectra for rhodamine B on ZnO nanostructures. (**a**) Spin-seeded ZnO nanorods. (**b**) Sputter-seeded ZnO nanorods. Each panel shows the illumination-corrected comparative spectra for the SERS effect of (black curve) Rh B on ZnO nanorods and (blue curve) rhodamine B (RhB) on ZnO nanorods deposited with 200 nm gold. Dark lines indicate average signal, while pastel regions indicate one standard deviation of measurement variance.

The Raman spectra of RhB deposited on spin-seeded and sputter-seeded ZnO-Au SERS substrates are shown when compared to their bare ZnO equivalents in Figure 6. Using the model analyte RhB, the SERS EF of spin-seeded ZnO substrates was compared to that of sputter-seeded ZnO substrates in three bands. Compared to an uncoated ZnO substrate, at 1358 cm^−1^, 1507 cm^−1^, and 620 cm^−1^, respectively, the spin-seeded structure showed Raman EFs of 1051.4, 979.52, and 721.49, while the sputter-seeded substrate showed Raman EFs of 1305.43, 1265.7, and 1041.04. Thus, the average Raman EF can be estimated at 917.47 for the spin-seeded substrate, and 1204.06 for the sputter-seeded substrate, and the effect of spin seeding is to decrease the EF by only 24%.

## 4. Discussion

In the present work, a low-cost procedure was used to achieve the seeded growth of ZnO nanorods on Si substrates by spin-coating; this offers several practical advantages over the previously demonstrated RF sputtering method. In addition to enabling smooth (uniform) seed deposition, wet processes such as spin-coating can be performed quickly at room temperature and pressure and require a lower level of dust control. For example, spin-coating for the deposition of organic photoresists in semiconductor manufacturing has been shown to be highly scalable [28].

As seen in Figure 2 and Figure 3, the change in the seeding technique significantly modulates the subsequent growth of ZnO nanorods. Compared to nanorods grown from sputtered seeds, spin-seeded nanorods show a greater variety of diffraction planes in XRD, demonstrating an increase in the variety of nanowire angles relative to the normal substrate. This disordered self-assembly is likely due to a change from the single-crystal substrate of the sputtered ZnO, which provides highly uniformly oriented nucleation sites, to a poly-crystalline seed structure in which each single-crystal seed provides its own single crystal for determining growth direction. Although each seed is deposited on the silicon surface in a random orientation during spin-coating, seeds are loosely bound to the surface during the low-temperature annealing step, resulting in a restricted set of potential orientations for crystal nucleation.

The previous literature on the hydrothermal synthesis process for preparing ZnO nanorods has shown that the ZnO nanorod diameter varies with the source of Zn in the deposition solution. Our results, as seen in Figure 3, show typical nanorod diameters of 40 to 50 nm, which is significantly larger than the 20 nm sizes obtained in the synthesis from nitrate solutions [29]. However, the observed diameters are consistent with some previous nitrate-based syntheses [6,19,20], and some of the shorter nanorods visible in Figure 3 do show narrow diameters of around 20 nm. This discrepancy with the literature may be related to the length of the rod grown during hydrothermal synthesis. Although not very significant for the diagnostic applications of SERS, further analysis into this discrepancy may lead to deeper insights into the difficult-to-probe kinetics of hydrothermal nanorod synthesis.

Monte Carlo simulation of extended SERS structures has previously been performed, although not recognized as such. Fraire et al. performed generalized Mie scattering simulations for 2D nanospheres using randomized clustering with a variety of polarizations, and with randomly assigned gaps in the SERS nano-clusters [9]. However, these structures were formed of relatively simple spherical components. By applying the COMSOL FEM platform rather than the more accurate generalized Mie theory, it is possible to simulate arbitrary domains, contingent on the availability of computational resources. Examples of these simulation domains are shown in Figure 5a,b.

It was rigorously shown by Le Ru and Etchegoin that the actual magnitude of the EF for Raman-generated dipoles can be approximated as being proportional to |E|^4^ when the dependence upon polarization components is averaged [10]. Although electrostatic simulations of SERS dynamics are known to significantly overestimate EF values [8], and FEM is only an approximate replacement for generalized Mie scattering [30], the Monte Carlo FEM simulations (the results of which are shown in Figure 5) predict a mean EF of 5.86 and 8.44 in the spin-coated and sputtered substrates, respectively. This corresponds to a predicted drop in the Raman EF of 1.44× in the spin-seeded substrate. Similarly, pairwise FEM simulations (Figure 4c) predict respective EFs of 4.41 to 11.12, corresponding to a predicted drop in the Raman EF of 2.5× in the spin-seeded samples. Although the magnitude of the simulated EF differs significantly from that seen experimentally, this is likely due to exaggeration of the electric fields in the “bare” substrate simulations, which are particularly sensitive to analyte refractive index. Importantly, similar effect magnitudes are seen in both the pairwise simulation and the full Monte Carlo simulation, and the same effects are seen experimentally.

Experimentally, it was observed that the variance in surface-averaged EF between the spin-seeded and sputter-seeded ZnO samples was only by a factor of 1.3 (Figure 6), with average EFs of 917.47 and 1204.06, respectively. For the sputter-seeded ZnO sample, the variance in the EF over the measured points was less than 10%, which provides a measure of the deviation, which may be attributed to the non-uniform drying of the test analyte. The spin-seeded sample had an observable standard deviation of ~19%, indicating that despite the higher randomness in nanorod orientation, the signal was relatively consistent over the substrate. These results demonstrate both the promise of FEM simulation for the modeling of complex SERS structures and the efficacy of the low-cost spin-seeded Au-coated ZnO nanorod substrate. A similar ratio of EF between the spin-seeded and sputter-seeded SERS substrates is observed when using a more traditional EF, defined as the total Raman intensity of the rhodamine-dropped SERS structures compared to that of RhB-coated bare Si substrates.

The previous literature on ZnO nanorods has asserted that high uniformity is likely necessary for clinical applications of ZnO-based SERS. Despite a severe lack of uniform rod orientation, we observe satisfactory SERS both in simulations and experimentally, with only a 1.3× EF difference between highly ordered surfaces and spin-seeded nanorod samples. While much less than the EFs of 10^8^ or higher observed for single-molecule SERS [8], the field EF of 917.5 is significant in comparison with other demonstrations of disordered substrates [13,14].

While the change in procedure from sputter seeding to spin-coating may appear minor and has clearly detrimental effects on Raman EF, the typical market pricing for the use of a shared sputtering evaporator is on the order of USD 200 per substrate due to limited capacity, inefficient use of seed material, large capital investments, and the requirement for vacuum. In contrast, spin-coating is a low-power benchtop procedure with much more efficient use of seed material and a tenfold increase in sample turnaround times due to the benchtop process. Based on present prices, and our experienced consumption rate of ZnO seed solution, we estimate the marginal cost of spin-seeded substrates at USD 0.20 per substrate. Amortizing the price of the spin-coater provides a roughly 100-fold improvement in cost per substrate deposited.

The low-cost fabrication of SERS substrates is likely to open up new applications for research and diagnosis. Taken together, the deleterious effect of disorder on the magnitude of the SERS enhancement for Au-coated ZnO nanorods prepared by spin-coated seed deposition was observed to be small. This is in contrast to the large economic benefit that may be realized by spin-coating on polycrystalline substrates, as opposed to more complex sputtering processes. Future research may combine these results with further low-cost substrates, such as paper [31] and plastic [32], or with alternative plasmonic materials [33] to minimize the cost of SERS in diagnosis.

## Figures and Tables

**Table 1:** Distribution of nanorod angles measured by XRD.

Diffraction Plane (*h k l*)	Tilt Angle from Normal	Solution Seed		Sputter Seed	
		2θ (°)	Proportion ^1^ (%)	2θ (°)	Proportion ^1^ (%)
(002)	0°	34.36	33.68	34.40	84.45
(101)	60°	36.20	23.27	-	-
(102)	41°	47.49	10.28	47.54	2.9
(110)	30°	56.52	10.78	-	-
(103)	72°	62.89	15.63	62.88	12.36
(112)	90°	67.95	6.36	67.91	0.29

^1^ Relative proportions are normalized to 100%.

## Appendix A

The following table shows the derived obscuration depth from cross-sectional FE-SEM, which was used to infer the number of nanorods to be simulated in 2D FEM and the nanorod density on the substrate.

**Table A1:** Obscuration of nanorods, as derived from Figure 3.

Depth of Occultation	Visible Nanorods
0	14
1	13
2	11
3	10
4	7
>4	~24
